# Characteristics of voriconazole-induced visual disturbances and hallucinations: case reports and literature review

**DOI:** 10.3389/fphar.2024.1420046

**Published:** 2024-11-07

**Authors:** Ya Liu, Ying Huang, Xiang Liu, Danxia Wang, Yixiang Hu

**Affiliations:** ^1^ Department of Clinical Pharmacy, Xiangtan Central Hospital (The Affiliated Hospital of Hunan University), Xiangtan, China; ^2^ Zhongshan Hospital of Traditional Chinese Medicine Afflilated to Guangzhou University of Chinese Medicine, Zhongshan, China; ^3^ Department of Pharmacy, Ningxiang People’s Hospital Affliated to Hunan University of Chinese Medicine, Changsha, China

**Keywords:** voriconazole, adverse reactions, CYP2C19 genotyping, clinical characteristics, management strategies

## Abstract

Voriconazole, a broad-spectrum antifungal agent, is considered the first-line treatment for invasive aspergillosis. In this article, we report three cases of patients who experienced visual disturbances and hallucinations following voriconazole therapy for invasive pulmonary aspergillosis. These symptoms appeared within 1 week after initiating voriconazole administration and resolved upon discontinuation or dose reduction of the drug. Considering the absence of any identifiable alternative cause and the temporal relationship with voriconazole initiation, these symptoms were attributed to the adverse effects of voriconazole. All three patients had trough concentrations exceeding 5 μg/mL at the time of adverse reactions, leading to subsequent therapeutic drug monitoring and dose adjustment. The clinical characteristics and management strategies of voriconazole-induced hallucinations and/or visual disturbances have been rarely reported previously. Therefore, our study reviewed and analyzed relevant case reports since 2014. This study highlights the importance of recognizing the potential risk of hallucinations and visual disturbances associated with voriconazole. Furthermore, our findings indicate that the route of voriconazole administration does not influence the frequency of these adverse events. Additionally, special attention should be given to monitoring adverse events related to voriconazole in Asian populations due to their higher prevalence of *CYP2C19* poor metabolizers. In the event of adverse reactions to voriconazole, diligent monitoring of therapeutic drug levels and dosage adjustments is crucial. These clinical characteristics and management strategies offer advantages in terms of enhancing drug efficacy, ensuring treatment continuity, and minimizing the incidence of other severe adverse reactions.

## Introduction

Voriconazole (VRC), a second-generation azole-antifungal agent, is the recommended initial treatment for serious fungal infections, including invasive pulmonary aspergillosis (Veringa et al., 2023; Wang et al., 2022). VRC is associated with various side effects such as fever, rash, gastrointestinal symptoms, headache, and hepatotoxicity (Boyd et al., 2004; de Almeida Campos et al., 2023; Kinoshita et al., 2011; Mohammed et al., 2022). However, there has been limited research conducted on the clinical characteristics and management strategies for hallucinations and/or visual impairments induced by voriconazole. In this study, we report three cases of patients who experienced visual disturbances and hallucinations following VRC treatment for invasive pulmonary aspergillosis. Additionally, we performed a comprehensive review and analysis of relevant case reports from the past decade to investigate the clinical characteristics and management strategies associated with these adverse reactions.

### Case presentation

#### Case 1

**FIGURE 1:**
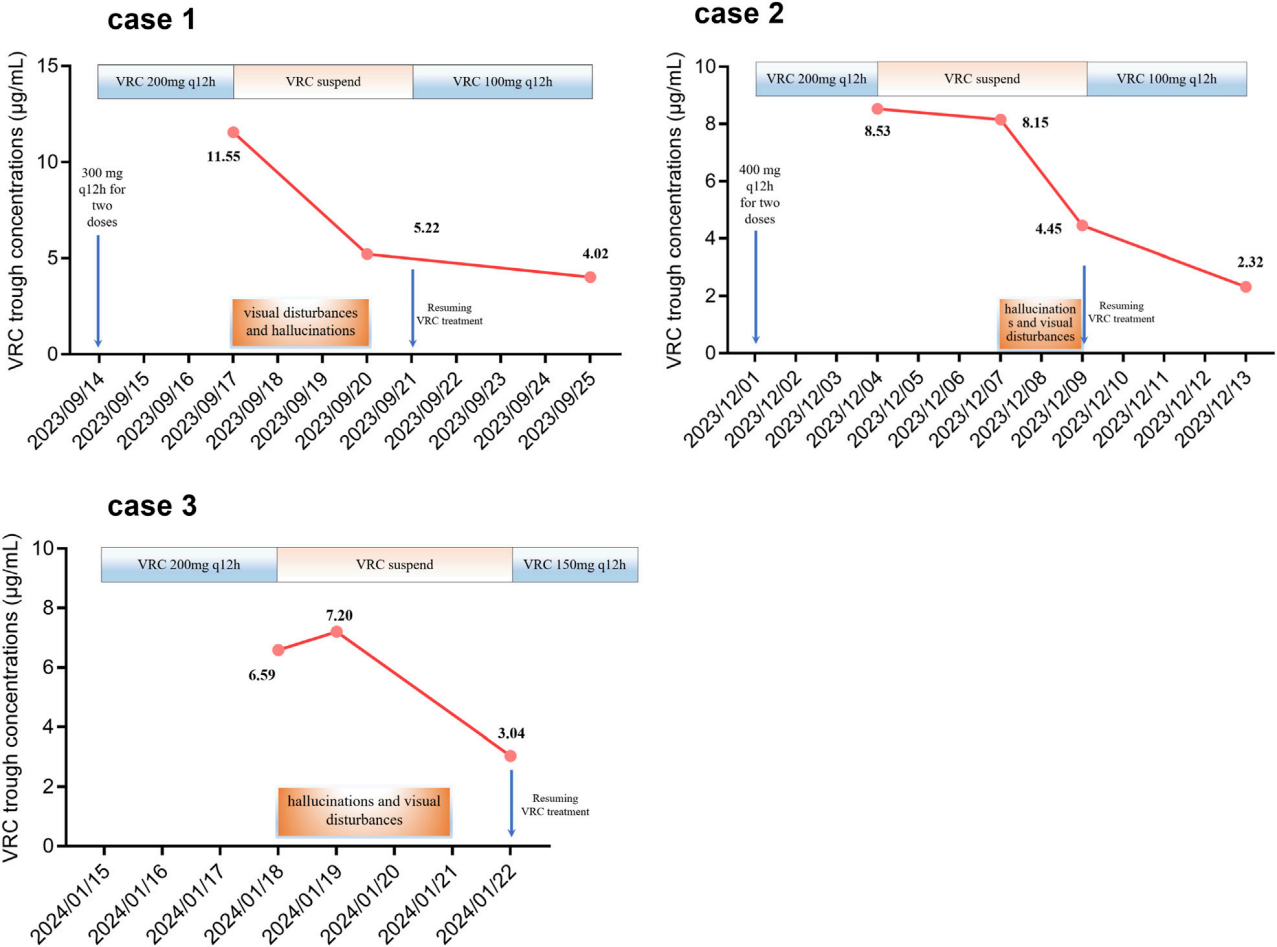
Alterations in the trough concentration of voriconazole and the occurrence of visual disturbances and hallucinations.

The patient, an 88-year-old male (with unknown body weight due to being bedridden), was admitted to our hospital with a history of “repeated cough and expectoration for over 20 years”. Subsequent physical examination revealed bilateral moist rales in the lungs, along with fever, abdominal pain, chest pain, and fecal incontinence. This patient did not have any concurrent neurological disorders. Chest computed tomography (CT) showed emphysema and infectious lung lesions. Serum levels of alanine aminotransferase (ALT), aspartate aminotransferase (AST), and creatinine (CREA) were 21.3 IU/L, 25.5 IU/L, and 94.0 μmol/L, respectively. Therefore, the patient received piperacillin sodium and tazobactam sodium for injection (4.5 g q12 h) along with aminophylline injection. Subsequently, Aspergillus was identified in sputum culture leading to a diagnosis of pulmonary aspergillosis. VRC tablets were initiated at a dose of 300 mg q12 h for two doses followed by maintenance therapy at a dose of 200 mg q12 h from September 14 onwards. On day four of VRC treatment, the patient experienced hallucinations and visual disturbances characterized by perceiving strange shadows in front of him along with red-green discoloration and symptoms of gibberish speech. On September 17, 2023, the trough concentration of VRC was measured to be elevated at 11.55 μg/mL, which correlated with the observed adverse effects including hallucinations and visual disturbances. Consequently, we decided to discontinue VRC administration due to its supratherapeutic level causing these adverse effects which completely resolved by September 20th when the trough concentration decreased to approximately normal levels, measuring at around 5.22 μg/mL simultaneously. VRC treatment was resumed on September 21st at a reduced dose regimen (100 mg q12 h). The subsequent trough concentration measurement on September 25th indicated therapeutic levels within range at approximately 4.02 μg/mL without recurrence of any VRC-induced adverse effects (Figure 1).

#### Case 2

An 85-year-old male (with unknown body weight due to being bedridden) with a medical history of COPD, bronchiectasis, diabetes, and coronary heart disease presented to our hospital. The patient has no prior neurological disorders. Subsequent tests revealed bilateral lower leg edema and bilateral moist rales in the lungs. A combination of acute exacerbation of COPD (AECOPD) and respiratory infection leading to respiratory failure was considered. Serum levels of ALT, AST, and CREA were 5.6 IU/L, 15.5 IU/L, and 157.0 μmol/L, respectively. Consequently, spironolactone tablets, meropenem for injection, and moxifloxacin injection were prescribed. Subsequently, the presence of serum galactomannan (GM) confirmed the diagnosis of pulmonary aspergillosis. VRC tablets were administered at a dose of 400 mg q12 h for two doses followed by 200 mg q12 h starting from December 1, 2023. On day four of VRC treatment, the trough concentration of VRC was observed to be elevated at 8.53 μg/mL. Subsequently, VRC administration was discontinued while the trough concentration levels were monitored. The patient experienced hallucinations and visual disturbances on December 7 which resolved by December 9 without intervention. The trough concentrations of VRC measured on December 7 and December 9 were 8.15 μg/mL and 4.45 μg/mL respectively. VRC treatment resumed on December 9 with a reduced dosage regimen (100 mg q12 h po). On December 13th, the trough concentration level reached a value of 2.32 μg/mL without any recurrence of adverse effects associated with VRC (Figure 1).

#### Case 3

An 74-year-old male (with unknown body weight due to being bedridden) was admitted to our hospital due to a history of recurrent cough. Digital X-ray photography (DX) revealed scattered speckled, flaky, and cable-like high-density shadows in the left middle and lower lung fields. The patient has no history of neurological disorders. Consequently, a diagnosis of chronic obstructive pulmonary disorder (COPD) with chronic inflammation of the left lung was considered. Additionally, the patient’s lung CT indicated bronchiectasis accompanied by infection. Serum levels of ALT, AST, and CREA were 26.6 IU/L, 20.9 IU/L, and 53.0 μmol/L, respectively. Consequently, methylprednisolone sodium succinate for injection, piperacillin sodium and tazobactam sodium for injection, and ambroxol hydrochloride injection were prescribed. Subsequently, the patient was found to be positive for Influenza A, and thus oseltamivir phosphate capsules were added. Specific IgG for Aspergillus fumigatus and positive GM levels were detected in the patient’s serum. Additionally, sputum culture confirmed the presence of Aspergillus fumigatus and Aspergillus flavus. Pulmonary aspergillosis was therefore diagnosed. VRC treatment at a dose of 200 mg q12 h commenced on January 15, 2024. On day 4 of VRC treatment, the patient experienced hallucinations and visual disturbances characterized by blurry vision and floating in front of his eyes. These symptoms appeared suddenly. Simultaneously, the trough concentration of VRC measured at 6.59 μg/mL raised concerns regarding drug-induced hallucinations and visual disturbances. Consequently, VRC administration was suspended while monitoring its trough concentration. Complete resolution of hallucinations and visual disorders occurred on January 21, 2024. The trough concentrations of VRC were 7.20 μg/mL on January 19th and decreased to 3.04 μg/mL on January 22nd respectively. VRC treatment resumed at a reduced dosage of 150 mg q12 h starting from January 22nd without recurrence of adverse effects induced by VRC (Figure 1).

## Discussion

We assessed our patient’s score on the Naranjo Scale (Naranjo et al., 1981). The three cases we report had respective weighted scores of 8, 5, and 5 on the Naranjo Scale, indicating that hallucinations and visual disturbances were probably caused by VRC rather than other medications. Then, we conducted an extensive search in both English and Chinese databases, including PubMed, Embase, Web of Science, Wanfang Data and the China National Knowledge Infrastructure (CNKI), for relevant literature published within the past 10 years using search terms such as “voriconazole”, “hallucinations”, “visual disturbances”, and “visual toxicity”. A comprehensive literature review was performed, which included a summary of eight studies investigating hallucinations and/or visual disturbances associated with VRC (Table 1) (Bayhan et al., 2016; Cao and Chen, 2020; Jansen et al., 2017; Jiang J., 2020; Kato et al., 2016; Shan and Rao, 2017; Zhang et al., 2021; Zheng et al., 2021). A total of fourteen patients were reported across the eight studies that were included. Of these, oral dosage form was administered to eight patients (57.1%), injectable dosage form to five patients (35.7%), and the mode of administration for one patient remained unknown (7.2%). Zonios et al. (2008) observed that hallucinations associated with the intravenous formulation vanish when VRC is administered orally. However, all three cases of VRC-induced visual disturbances and hallucinations reported in this article were orally administered. Therefore, we speculate that the route of VRC administration does not affect the frequency of hallucinations and visual disturbances.

**TABLE 1:** Literature review of visual disturbances and hallucinations caused by Voriconazole.

Authors/Year (References)	Country	Age (years)	Sex	Body weight (kg)	Diagnosis	Dose	Dosage form	Clinical symptoms	Onset time (days)	VRC trough concentration (µg/mL)	CYP2C19 genotype	Therapeutic adjustment
Jansen et al. (2017)	United States	67	Male	79.3	alcoholic hepatitis, chronic pancreatitis, COPD	400 mg q12 h for 2 doses, then 200 mg q12 h	oral	hallucinations	2	9.0	CYP 2C19*1/*2	VRC withdrawal
Kato et al. (2016)	Japan	68	Female	45	diabetes, cervical cancer	300 mg q12 h for 2 doses, then 200 mg q12 h	oral	hallucinations	4	NA	NA	VRC withdrawal and switch to Micafungin
Kato et al. (2016)	Japan	81	Male	50	diabetes, hypertension	300 mg q12 h for 2 doses, then 200 mg q12 h	oral	hallucinations	2	NA	NA	VRC withdrawal and switch to Itraconazole
Kato et al. (2016)	Japan	67	Male	46	diabetes, upper bile duct cancer	300 mg q12 h for 2 doses, then 200 mg q12 h	intravenous	hallucinations	2	3.79	NA	VRC continuation
Kato et al. (2016)	Japan	68	Female	31.5	pneumonia	200 mg q12 h for 2 doses, then 100 mg q12 h	NA	hallucinations	42	1.28	NA	VRC withdrawal and switch to Itraconazole
Kato et al. (2016)	Japan	76	Male	54.6	pneumonia, acute lymphoid leukemia	300 mg q12 h for 2 doses, then 200 mg q12 h	oral	visual disturbances	3	7.49	NA	VRC decrement
Kato et al. (2016)	Japan	15	Female	38.2	pulmonary aspergillosis	200 mg q12 h	oral	visual disturbances	9	4.45	NA	VRC decrement
Zheng et al. (2021)	China	9	Female	28	intermediate-risk T-cell (III) leukemia, sepsis	200 mg q12 h	oral	hallucinations and visual disturbance	2	3.6	NA	VRC withdrawal
Bayhan et al. (2016)	Turkey	16	Female	55	T-cell leukemia, invasive pulmonary aspergillosis	400 mg q12 h	intravenous	hallucinations and visual disturbance	4	NA	NA	VRC continuation
Shan and Rao (2017)	China	63	Male	NA	Lung infection	200 mg q12 h	intravenous	hallucinations	3	NA	NA	VRC withdrawal and switch to Caspofungin
Jiang (2020)	China	68	Male	NA	Broncholung cancer	200 mg q12 h	intravenous	hallucinations	1	NA	NA	VRC withdrawal and switch to Fluconazole
Zhang et al. (2021)	China	77	Male	NA	COPD	200 mg q12 h	oral	hallucinations and visual disturbance	2	NA	NA	VRC withdrawal
Zhang et al. (2021)	China	58	Male	NA	hemoptysis	200 mg q12 h	oral	hallucinations	3	NA	NA	VRC withdrawal
Cao and Chen (2020)	China	53	Female	52	Bronchiectasis with infection	300 mg q12 h for 2 doses, then 200 mg q12 h	intravenous	visual disturbance	5	5.7	CYP 2C19*1/*2	VRC decrement

VRC, voriconazole; COPD, chronic obstructive pulmonary disease; NA, not available.

Although the optimal therapeutic concentration of VRC remains unknown, it is generally recommended to fall within the range of 0.5–5.0 μg/mL (Chen et al., 2018). However, it is currently unclear whether there are variations in the concentration range of voriconazole in the blood of elderly individuals. A positive correlation has been identified between the concentration of VRC in the plasma and visual disturbances induced by the drug (Tan et al., 2006). Specifically, the odds ratio for visual disturbances increased by 4.7% for every 1 μg/mL increase in plasma VRC concentration. Furthermore, a notable discrepancy is apparent in the mean plasma VRC concentration (2.52 μg/mL) among 78 patients who did not experience hallucinations *versus* the 4.53 μg/mL level observed in 14 of 16 patients who reported hallucinations (Zonios et al., 2014). It was discovered that the individuals with plasma VRC concentrations >5 μg/mL [10/31 subjects (32%)] had a higher incidence of neurotoxic adverse effects, specifically visual and auditory hallucinations, compared to those with concentrations ≤5 μg/mL [2/170 patients (1.2%)] (Dolton et al., 2012). Of note, a study revealed a strong association between VRC-induced hallucinations and visual impairment (Imataki et al., 2008).

According to a systematic review and meta-analysis of 1,158 patients, VRC increased the risk of neurotoxicity when administered at a trough concentration exceeding 4.0 μg/mL (Jin et al., 2016). In the present study, we observed hallucinations and visual disturbances in three patients with a trough concentration of VRC >5 μg/mL (Figure 1). Consequently, we postulate that an elevated plasma concentration of VRC may be associated with an increased susceptibility to hallucinations and visual disturbances. However, a retrospective analysis of 103 patients revealed no statistically significant difference in plasma VRC concentrations between individuals with and without hallucinations (Sakurada et al., 2016). Further investigations are warranted to determine whether the occurrence of visual disturbances and hallucinations depends on VRC concentration.

The hepatic enzyme CYP2C19 plays a crucial role in the metabolism of VRC, with genetic polymorphisms significantly influencing its metabolic process and leading to substantial interindividual variations in plasma concentrations of this drug (Lamoureux et al., 2016). The most prevalent loss-of-function allele for *CYP2C19* is *2, with frequencies of approximately 15% among Caucasians and Africans, and 29%–35% among Asians. Poor metabolizers of *CYP2C19* account for around 2%–5% in Caucasians and Africans, while reaching approximately 15% in Asians (Scott et al., 2011). Among the reviewed articles, there were a total of 14 patients from Japan (6 cases, 42.9%), China (6 cases, 42.9%), Turkey (1 case, 7.1%), and the USA (1 case, 7.1%). Notably, all three documented cases of VRC -induced adverse reactions occurred within the Chinese population. Therefore, it is essential to pay further attention to adverse reactions associated with VRC use in Asians and consider implementing therapeutic drug monitoring as well as *CYP2C19* genotyping specifically within Asian populations.

The management of pulmonary aspergillosis typically requires long-term treatment, and abruptly discontinuing or altering medications may potentially impact the efficacy of antifungal therapy to some extent. Moreover, compared to alternative antifungal agents, VRC is a more favorable choice for outpatients undergoing persistent treatment due to its dosage form and price. Among the 14 patients reviewed in this study, when adverse reactions such as visual impairment and hallucination occurred due to VRC, four patients (28.6%) discontinued the medication, three patients (21.4%) reduced the dosage, two patients (14.3%) maintained the original regimen (One patient exhibited a trough concentration of 3.79 for VRC while no data was available for the other patient), and five patients (35.7%) switched to alternative antifungal drugs (e.g., fluconazole, itraconazole, micafungin or caspofungin). Furthermore, in the three cases of VRC -induced visual disturbances and hallucinations documented in this study, all patients exhibited a trough concentration exceeding 5 μg/mL. Subsequently, therapeutic drug monitoring was implemented and subsequently resulted in dose reduction leading to resolution of these adverse reactions. Hence, we suggest that the implementation of therapeutic drug monitoring in conjunction with careful dosage adjustment represents a beneficial strategy for alleviating the visual disturbances and hallucinations induced by VRC.

This study has the following limitations. Firstly, it is constrained in estimating event incidence as it based on available cases and literature reviews. Future research will require a greater reliance on real-world data and individual case reports to further elucidate the clinical features of visual disturbances induced by voriconazole. Secondly, the search was limited to electronic databases, resulting in the unavailability of full texts for some studies, potentially introducing bias in selection and information. Furthermore, the included cases were from various clinical settings with incomplete crucial information. Discrepancies in diagnostic methods, medical protocols, and reporting standards may have influenced the data, implying inherent bias in this article. Thirdly, these three cases lack some clinical information, such as weight data and CYP2C19 genetic polymorphism. Increasing evidence implicates that the visual disturbances and hallucinations induced by VRC may be associated with individualized factors, such as CYP2C19 genetic polymorphism and drug sensitivity. Additionally, patient 3 was not administered the initial loading dose but was directly given the maintenance dose. Nevertheless, this study conducted a preliminary investigation into the clinical features of visual disturbances and hallucinations caused by VRC, aiming to enhance understanding and management of the adverse reactions associated with VRC.

## Conclusion

In summary, the occurrence of visual disturbances and hallucinations induced by VRC is frequently observed in clinical settings. Patients undergoing VRC treatment should remain vigilant for potential symptoms such as blurred vision, altered color perception, visual and auditory hallucinations, among others. If any of these symptoms manifest during VRC administration, therapeutic drug monitoring is recommended along with appropriate dosage adjustments if necessary. Regular follow-up assessments should be conducted throughout the treatment period. Additionally, clinicians should promptly identify visual disturbances and hallucinations caused by VRC to enable timely intervention.

## Data Availability

The original contributions presented in the study are included in the article/supplementary material, further inquiries can be directed to the corresponding author.

## References

[B1] Bayhan G. I. Garipardic M. Karaman K. Akbayram S. (2016). Voriconazole-associated visual disturbances and hallucinations. Cutan. Ocul. Toxicol. 35 (1), 80–82. 10.3109/15569527.2015.1020544 25799212

[B2] Boyd A. E. Modi S. Howard S. J. Moore C. B. Keevil B. G. Denning D. W. (2004). Adverse reactions to voriconazole. Clin. Infect. Dis. 39 (8), 1241–1244. 10.1086/424662 15486850

[B3] Cao J. Chen X. (2020). Analysis and treatment of visual abnormality caused by voriconazole gene polymorphism: a case report. Cent. South Pharm. 18 (7), 1257–1258. 10.7539/j.issn.1672-2981.2020.07.038

[B4] Chen K. Zhang X. Ke X. Du G. Yang K. Zhai S. (2018). Individualized medication of voriconazole: a practice guideline of the division of therapeutic drug monitoring, Chinese pharmacological society. Ther. Drug Monit. 40 (6), 663–674. 10.1097/ftd.0000000000000561 30192314 PMC6250289

[B5] de Almeida Campos L. Fin M. T. Santos K. S. de Lima Gualque M. W. Freire Cabral A. K. L. Khalil N. M. (2023). Nanotechnology-based approaches for voriconazole delivery applied to invasive fungal infections. Pharmaceutics 15 (1), 266. 10.3390/pharmaceutics15010266 36678893 PMC9863752

[B6] Dolton M. J. Ray J. E. Chen S. C. Ng K. Pont L. G. McLachlan A. J. (2012). Multicenter study of voriconazole pharmacokinetics and therapeutic drug monitoring. Antimicrob. Agents Chemother. 56 (9), 4793–4799. 10.1128/aac.00626-12 22751544 PMC3421881

[B7] Imataki O. Ohnishi H. Kitanaka A. Kubota Y. Ishida T. Tanaka T. (2008). Visual disturbance comorbid with hallucination caused by voriconazole in the Japanese population. Int. J. Hematol. 88 (1), 3–6. 10.1007/s12185-008-0114-3 18574651

[B8] Jansen J. W. Sen S. K. Moenster R. P. (2017). Elevated voriconazole level associated with hallucinations and suicidal ideation: a case report. Open Forum Infect. Dis. 4 (1), ofw215. 10.1093/ofid/ofw215 28480228 PMC5414099

[B9] Jiang J. (2020). Voriconazole induced hallucination: a case report. Her. Med. 39 (1), 120–121. 10.3870/j.issn.1004-0781.2020.01.026

[B10] Jin H. Wang T. Falcione B. A. Olsen K. M. Chen K. Tang H. (2016). Trough concentration of voriconazole and its relationship with efficacy and safety: a systematic review and meta-analysis. J. Antimicrob. Chemother. 71 (7), 1772–1785. 10.1093/jac/dkw045 26968880 PMC4896404

[B11] Kato H. Hagihara M. Hamada Y. Koizumi Y. Nishiyama N. Yamagishi Y. (2016). Visual disturbance or central symptom like hallucination in patients treated voriconazole: report of six cases. Jpn. J. Antibiot. 69 (3), 143–150. 30226950

[B12] Kinoshita J. Iwata N. Ohba M. Kimotsuki T. Yasuda M. (2011). Mechanism of voriconazole-induced transient visual disturbance: reversible dysfunction of retinal ON-bipolar cells in monkeys. Invest. Ophthalmol. Vis. Sci. 52 (8), 5058–5063. 10.1167/iovs.11-7183 21436272

[B13] Lamoureux F. Duflot T. Woillard J. B. Metsu D. Pereira T. Compagnon P. (2016). Impact of CYP2C19 genetic polymorphisms on voriconazole dosing and exposure in adult patients with invasive fungal infections. Int. J. Antimicrob. Agents 47 (2), 124–131. 10.1016/j.ijantimicag.2015.12.003 26775563

[B14] Mohammed Y. Abousamra A. Abdeldayem A. A. I. Zafar M. Muhammad T. (2022). Voriconazole-induced cholestatic hepatotoxicity in an immune competent patient. Cureus 14 (1), e21346. 10.7759/cureus.21346 35103217 PMC8769073

[B15] Naranjo C. A. Busto U. Sellers E. M. Sandor P. Ruiz I. Roberts E. A. (1981). A method for estimating the probability of adverse drug reactions. Clin. Pharmacol. Ther. 30 (2), 239–245. 10.1038/clpt.1981.154 7249508

[B16] Sakurada H. Yasuhara K. Kato K. Asano S. Yoshida M. Yamamura M. (2016). An investigation of visual hallucinations associated with voriconazole administration to patients with hematological malignancies. Pharmazie 71 (11), 660–664. 10.1691/ph.2016.6725 29441972

[B17] Scott S. A. Sangkuhl K. Gardner E. E. Stein C. M. Hulot J. S. Johnson J. A. (2011). Clinical Pharmacogenetics Implementation Consortium guidelines for cytochrome P450-2C19 (CYP2C19) genotype and clopidogrel therapy. Clin. Pharmacol. Ther. 90 (2), 328–332. 10.1038/clpt.2011.132 21716271 PMC3234301

[B18] Shan W. Rao Y. (2017). Voriconazole injection induced phantom in patient with pulmonary infection opsia: a case report. Chin. Hosp. Pharm. J. 37 (8), 781–782. 10.13286/j.cnki.chinhosppharma-cyj.2017.08.25

[B19] Tan K. Brayshaw N. Tomaszewski K. Troke P. Wood N. (2006). Investigation of the potential relationships between plasma voriconazole concentrations and visual adverse events or liver function test abnormalities. J. Clin. Pharmacol. 46 (2), 235–243. 10.1177/0091270005283837 16432276

[B20] Veringa A. Brüggemann R. J. Span L. F. R. Biemond B. J. de Boer M. G. J. van den Heuvel E. R. (2023). Therapeutic drug monitoring-guided treatment versus standard dosing of voriconazole for invasive aspergillosis in haematological patients: a multicentre, prospective, cluster randomised, crossover clinical trial. Int. J. Antimicrob. Agents 61 (2), 106711. 10.1016/j.ijantimicag.2023.106711 36642232

[B21] Wang T. Miao L. Shao H. Wei X. Yan M. Zuo X. (2022). Voriconazole therapeutic drug monitoring and hepatotoxicity in critically ill patients: a nationwide multi-centre retrospective study. Int. J. Antimicrob. Agents 60 (5-6), 106692. 10.1016/j.ijantimicag.2022.106692 36372345

[B22] Zhang X. Yang H. Guo X. Xie P. (2021). Adverse reactions of visual disturbances and hallucinations induced by voriconazole tablets in two cases and related literature review. Chin. J. Pharmacovigil. 18 (6), 588–591. 10.19803/j.1672-8629.2021.06.20

[B23] Zheng R. Li Y. Guo C. Pei Y. Ke Z. Huang L. (2021). Voriconazole induced hallucinations and visual disturbances in a female child: a case report and literature review. Front. Pediatr. 9, 655327. 10.3389/fped.2021.655327 33968855 PMC8102700

[B24] Zonios D. Yamazaki H. Murayama N. Natarajan V. Palmore T. Childs R. (2014). Voriconazole metabolism, toxicity, and the effect of cytochrome P450 2C19 genotype. J. Infect. Dis. 209 (12), 1941–1948. 10.1093/infdis/jiu017 24403552 PMC4038142

[B25] Zonios D. I. Gea-Banacloche J. Childs R. Bennett J. E. (2008). Hallucinations during voriconazole therapy. Clin. Infect. Dis. 47 (1), e7–e10. 10.1086/588844 18491963 PMC2727751

